# Clinical Report of a Compound Comminuted Fracture, Etc.

**Published:** 1882-08-20

**Authors:** 

**Affiliations:** Marianna, Ark.; Marianna, Ark.


### Southern Medical Record:

EDITORS:

T. S. POWELL, M.D. W. T. GOLDSMITH, M.D. R. C. WORD, M.D.

*R. C. WORD, M.D., Managing Editor.*

☞All Communications and Letters on Business connected with the RECORD must be addressed to the Managing Editor.

VOL. XII. ATLANTA, GA., AUGUST 20, 1882. No. 8.

### ORIGINAL AND SELECTED ARTICLES.

### CLINICAL REPORT

*Of a Compound Comminuted Complicated Fracture of the Superior Maxilla, and Compound Complicated Fracture of the Inferior Maxilla, Successfully Treated.*

BY DRS. HAYES BROS., MARIANNA, ARK.

Read before the Lee County Medical Association, at the June Meeting, by W. G. Hayes, M. D.

Maj. C——, æt. 52, married, a planter, was a subject of chronic diarrhœa while in the army; except with threatening attacks of it since from time to time, health has been otherwise good.

Gentlemen: As this case is a peculiar one and nothing like it has attracted my attention, either in the authorities or journals, I am permitted to hope that a brief history of it will be neither uninteresting nor uninstructive.

Dec. 18th, 1881. Sunday evening at 3 o'clock we were called in great haste to go five miles in the country to see Maj. C——, who had been thrown from a buggy by a runaway team, upsetting the buggy and throwing the two occupants out.

The Major was thrown with great force against the end of a fence rail, which came in contact with the right side of the face, was taken up insensible and carried to a neighboring house, where we found him one hour and a half afterwards. He complained of our delay, and thought we had put him off too long. We found him covered with blood, his face badly mangled and swollen, his mouth clotted, his speech very imperfect, and the blood running anteriorly and posteriorly from the nose.

It was a horrible sight to witness, and distressing in prognosis. After removing the blood from the face, we proceeded by trimming close the beard. On first sight we found two gashes in the cheek, each about two and a half inches long and extending to the bone; one beginning directly beneath the right nostril and extending irregularly to a point about the centre of the cheek; the other divided the lower lip half way between the centre and the right corner of the mouth, extending downward and to the right.

After a brief but satisfactory examination (under sponge and water) of the fractured jaws, we promptly closed the lacerations by stitches.

The right cheek was badly complicated, the superior maxilla being comminuted—also the malar on the same side. The teeth on this side were easily moved separately, and were held in position with the crushed bone only by the gusm, which were not lacerated.

The gash made in the laceration of the Levator muscles also severed branches from the mouth and nose, and soon he vomited a large amount from the stomach, and was relieved of the nausea that he had been complaining of, and then rested quietly under a dose of chloral until we could get an ambulance to take him home.

On arrival, at 8 o'clock, p. m., had a free natural action from the bowels; was put to bed with pulse feeble and breathing difficult, which was due, in part, to clotted blood in the nose, that we would not remove for fear of fresh hemorrhage.

Treatment as indicated, whisky and carb. ammonia, chloral hydrate and warm appliances; bowels acting again at 11 o'clock, free, bilious stool; quite restless all night.

Monday, 19th. Deglutition difficult this morning—due to swelling of soft parts and complication of fracture; spitting or blowing out large quantities of coagulum. At 10 o'clock an action from the bowels, all blood, and due to a deposit which had been accumulating in the stomach since 5 o'clock the evening before, the time at which he vomited freely.

In consideration of the complications in this case, Dr. W. B. Rogers, of Memphis, was called, who met us at this date; and on close examination (the coagulum having passed from the mouth and the patient well rested) he discovered a fracture, also, on the left side, in the superior maxilla, passing transversely across the facial surface, near the canine fossa, and downward in front between the canine and incisive teeth.

The consideration in this council (in which Dr Sumpter was invited to take part) was, first, the demand for an operation to take away all comminuted bone from the right cheek.

Second, the possible repair of bone, if properly adjusted and supported in a vulcanized rubber plate, to be prepared by a skillful surgeon dentist.

Yet, age and condition, long and tedious treatment, and doubtful repair were opposing features in this plan. It was nevertheless agreed upon, though reluctantly; yet we never had reason afterward to regret it.

Treatment: Bitter wine of iron, dose ℥ss. ter die; chloral hydrate, pro re nata. Listerine as a disinfectant and antiseptic. Nose and mouth syringed out frequently, with much discharge of clots and muco-pus.

Tuesday, 20th. Rested well last night; is now taking beef tea, maltine and cream. Listerine used freely to mouth and nose; breathing some better; deglutition improved on account of bandage being tightened, and supporting action of jaws. Was examined by Dr. N. N. Hayes, dentist, of Helena, who discovered still another fracture that had escaped our notice up to this time (although it had been suspected). This was a fracture of the shaft of the inferior maxilla, just in front of the mental foramen of the right side, which corresponded with the gash of the lower lip that has already been described. The discovery of this fracture very materially increased the difficulty in our plan of treatment. We were now deprived of the leverage and support of the lower jaw; and it was necessary to contrive a plate, so as to act as a stay, both to the upper and lower jaws. The patient was anæsthetized and the impression taken.

Wednesday, 21st. Rested badly last night; troubled by displacement of bandages; swallowing difficult; patient low-spirited and fearful of being strangled. Temperature having been very little above normal up to this time, is now exaggerated into fever.

Thursday, 22d. Seems better this morning, taking nourishment and tonic with more ease; is troubled about the adjusting of the plate that is to be put in the mouth this morning. The dentist who had been laboring constantly and skillfully for two days, was now ready. The plate, which is before me for inspection, has two surfaces—an upper and lower, with sockets fitted for the stay of upper and lower teeth when the parts of fracture are evenly adjusted, and resembles somewhat a long piece of "popping" wax or chewing-gum that a girl would pull from her mouth, "V" shaped, showing the impression of every tooth in the head. You may observe that on the lower margin of this plate there is a corresponding gap to the one made in the lower jaw by the evacuation of the five teeth we have already described; this gap in the plate was made as large as possible, for through it only could we have ingress to the mouth.

The plate was introduced by the dentist, and as the intelligent assistance of the patient was needed in placing it, he was not anæsthetized, but was compelled to endure the adjustment of fracture and setting teeth in the plate with much pain, which he did with more complacency than we expected.

The skillful arrangement of this plate adapted itself to the jaws, approximating a natural position. After applying our bandages and an isinglass plaster passed across the chin to carry off the fluids from the mouth, he was permitted to rest.

Through the gap described, we were able to pass the nozzle of a syringe, and with antiseptic solutions his mouth and nostrils were frequently cleansed; and a tablespoon could enter sufficiently to turn in its liquid contents. The treatment as already given, was kept up with very little variation for two months.

On the eighth day there was a rise of temperature, with a more liberal discharge of muco-pus, and much complaint of pain in the jaws. This high inflammatory action lasted until the 13th day, when it began to subside. At this time, being the 1st day of January, we removed bandages with much care, cleansed and redressed the face, with intention to let the plate remain in situ for a few more days, but next day found plate entirely dislodged, after a spell of coughing, which was produced by imperfect deglutition. The plate was then removed, dressings re-applied, and after taking two stitches in the hair-lip that was forming from the gash below, we congratulated our patient that union was rapidly being completed in the fractured parts.

Feb. 1st. He began to eat soft eggs, rice, gelatine and other semi-solids. At this time a small spicula of bone was taken from the mouth, and an abscess formed beneath the fracture of the lower jaw. This abscess was kept open for three months as a drainage; one small spicula passed in the meantime.

Four months after injury the light bandage was taken off. The beard having grown out sufficiently, leaves no deformity when the mouth is closed, except the cicatrix across the levator muscles. The action of these muscles and sensitive nerves are left imperfect, but are improving by use. He can now eat any well prepared food; health good; attentive to business, and is grateful for the results in the case.

